# HBV and HIV viral load but not microbial translocation or immune activation are associated with liver fibrosis among patients in South Africa

**DOI:** 10.1186/s12879-018-3115-8

**Published:** 2018-05-08

**Authors:** Tongai Gibson Maponga, Monique I. Andersson, Christoffel J. van Rensburg, Joop E. Arends, Jantjie Taljaard, Wolfgang Preiser, Richard H. Glashoff

**Affiliations:** 1 Division of Medical Virology, Stellenbosch University Faculty of Medicine and Health Sciences, Tygerberg, Cape Town South Africa; 2 Oxford University Hospitals NHS Foundation Trust, Oxford, United Kingdom; 3 Division of Gastroenterology, Stellenbosch University Faculty of Medicine and Health Sciences, Tygeberg, Cape Town South Africa; 4 Department of Internal Medicine, Section Infectious Diseases, University Medical Center Utrecht (UMCU), Utrecht University, Utrecht, The Netherlands; 5 Division of Infectious Diseases, Stellenbosch University Faculty of Medicine and Health Sciences, Tygerberg, Cape Town South Africa; 6 Division of Medical Microbiology & Immunology, Stellenbosch University Faculty of Medicine and Health Sciences & National Health Laboratory Service, Tygerberg, Cape Town South Africa

**Keywords:** Hepatitis B infection, HIV, Transient elastography, Cytokines, Anti-retroviral treatment, Viraemia, Microbial translocation products

## Abstract

**Background:**

Co-infection with HIV negatively impacts the progression of chronic hepatitis B virus (HBV) infection, including causing rapid progression to liver fibrosis. Sub-Saharan Africa represents arguably the most important intersection of high endemicity of both chronic hepatitis B virus (HBV) infection and HIV infection.

**Methods:**

We recruited 46 HBV/HIV-co-infected; 47 HBV-monoinfected; 39 HIV-monoinfected; and 37 HBV/HIV-uninfected patients from Tygerberg Hospital, Cape Town, South Africa. All HIV-infected patients were on antiretroviral therapy for ≥3 months. Liver stiffness measurements were assessed using the Fibroscan (Fibroscan 402, Echosens). Cell-based immunomarkers were measured by flow cytometry. Soluble serum/plasma immunomarkers were measured by Luminex technology and enzyme immunoassays. HIV (COBAS/Ampliprep TaqMan HIV-1) and HBV viral loads (in-house assay) were also performed.

**Results:**

HBV/HIV co-infected patients showed significantly higher levels of immune activation %CD8+/HLA-DR+/CD38+ (median 30%, interquartile range: 17–53) and %CD8+/PD-1 (median 22%, interquartile range: 15–33), *p* ≤ 0.01 compared to all other study groups. Despite this, the HBV-mono-infected group had the highest proportion of patients with advanced liver fibrosis (≥13 kPa) as measured by Fibroscan (18%). HBV mono-infected patients showed highest expression of most cytokines including IL-17 and basic fibroblastic growth factor. There was significant positive correlation between detectable HIV and HBV viral replication and liver fibrosis but not immune activation or gut translocation.

**Discussion:**

Highly-active antiretroviral therapy, including tenofovir, is effective against both HIV and HBV. Earlier therapy in the co-infected patients may therefore have controlled viral replication leading to better fibrosis scores when compared to HBV mono-infection in this study. On-going HBV and HIV viraemia, rather than microbial translocation or immune activation, appear to be the drivers of liver fibrosis. Moderate to advanced liver fibrosis in HBV-mono-infection may well indicate poor access to screening and treatment of HBV infection.

**Electronic supplementary material:**

The online version of this article (10.1186/s12879-018-3115-8) contains supplementary material, which is available to authorized users.

## Background

Sub-Saharan Africa represents arguably the most important intersection of high endemicity of both chronic hepatitis B virus (HBV) infection and HIV infection. HIV infection has been reported to alter the natural history of HBV by increasing HBV replication rates leading to elevated hepatitis B viraemia, delaying seroconversion from HBeAg to anti-HBe and also hastening the development of liver fibrosis [1]. Findings from elsewhere have shown that patients co-infected with HIV and HBV present with rapidly progressive liver disease, have a higher risk of developing cirrhosis and an elevated risk of death from liver-related disease compared to those with either HBV or HIV infection alone [2–5].

Chronic HBV infection is associated with immune tolerance in the liver, particularly during the immune tolerance phase [6]. However, HBV/HIV co-infection may lead to the loss of immune tolerance. Acute HIV infection results in severe depletion of gut associated lymphoid tissue cells (primarily memory T lymphocytes), which is associated with epithelial damage and results in leakage of gut-derived microbial antigens into the systemic circulation. Consequently, individuals with HIV infection have significantly elevated systemic levels of microbial products compared to HIV-uninfected individuals [7]. Translocated microbial products cause immune activation and have been associated with hepatic fibrosis [8]. Increased serum LPS levels are associated with liver fibrosis progression from various aetiologies such as chronic misuse of alcohol and non-alcoholic steatohepatitis [9].

Within the liver environment, LPS binds to TLR-4 on Kupffer cells and hepatocytes [10]. The interaction of LPS with TLR-4 initiates secretion of proinflammatory and fibrogenic cytokines [9, 11, 12]. Several pro-inflammatory cytokines, such as IL-1β, IL-6 and IFN-γ, have been associated with chronic inflammation in the liver environment and may be important in the relationship between inflammation and hepatocarcinogenesis [13]. The expression of these pro-inflammatory cytokines has been shown to increase with exposure to LPS while secretion of potentially protective anti-inflammatory cytokines such as IL-10 is reduced [13]. We explored the association between immune dysregulation caused by HIV infection and development of liver fibrosis in South African patients with or without HBV co-infection with the hypothesis that in patients with HIV/HBV co-infection, HIV facilitates the development of liver fibrosis via immune activation in response to the leakage of gut microbial products into the systemic circulation.

## Methods

### Study participants

Ethics approval was obtained from the Health Research Ethics Committee at Stellenbosch University. HBV/HIV co-infected and HIV-mono-infected patients were recruited from the Infectious Diseases Clinic while HBV-mono-infected and HBV-/HIV-uninfected patients (controls) were enrolled among patients attending the Gastroenterology Clinic at Tygerberg Hospital, Cape Town following written informed consent. Patients with other known liver diseases or HCV infection were excluded from the study. Patients of age < 18 years were also excluded. Epidemiological data as well as data on alcohol consumption and history of herbal medicine use were collected using a study-specific questionnaire.

### Non-invasive measurement of liver fibrosis and specimen collection

Patients had liver stiffness measurements by transient elastometry (TE) using the Fibroscan 402 (Echosens, Paris, France). TE cut-off values of < 7 kPa, 7.0–13.0 and ≥ 13 kPa were used to assign liver fibrosis severity as defined previously [14]. Blood was drawn for platelet count, aspartate transaminase (AST) and alanine transaminase (ALT) used to calculate the AST to platelet ratio index (APRI) and fibrosis-4 (FIB-4) scores. Aliquots of EDTA whole blood were also prepared for flow cytometric immune cell phenotyping.

### Virological and immunological testing

Serum specimens of HBV-infected patients were tested for HBeAg and antibodies to HBeAg (anti-HBe) using DiaSorin ETI-EBK PLUS and ETI-AB-EBKPLUS immunoassay kits (DiaSorin, Saluggia, Italy). HBV DNA testing was performed using an in-house quantitative PCR as previously described [15]. Samples with detectable HBV DNA were sequenced on the polymerase region of the HBV genome. HIV-1 viral loads were performed using the COBAS/Ampliprep TaqMan HIV-1 version 2.0 on the COBAS TaqMan Analyser (Roche Molecular Systems, CA). CD4 cell counts were performed on EDTA-anticoagulated blood using the BD TruCount on the BD FACSCalibur instrument (BD Biosciences, San Jose, CA).

### Flow cytometric measurement of % CD8+ and CD4+ T-cells expressing CD38, HLA-DR, PD-1 and CTLA-4

Two panels were designed; Immune Activation Panel and Immune Exhaustion Panel. The Immune Activation Panel consisted of the following fluorochrome-coupled monoclonal antibodies: CD3-FITC; CD4-ECD; CD8-PC-7; CD45-KO; CD38-PE and; HLA-DR-APC (Beckman Coulter, MI). The Immune Exhaustion Panel was composed of CD3-FITC; CD4-ECD; CD8-PC7; CD45-KO; CTLA-4-PE and; PD-1-APC. Flow cytometry data acquisition was performed using a Navios flow cytometer (Beckman Coulter). Optimal volatges for acquisition were determined, compensation and fluorescence minus one analyses were performed before testing of study samples. The gating strategy is shown in Additional file 1: Figure S1. Data analysis was performed using Beckman Coulter Kaluza software.

### Measurement of serum markers of microbial translocation and soluble cytokines

Plasma soluble CD14 (sCD14) and lipoprotein binding protein (LBP) levels were measured as biomarkers of LPS activity by enzyme immunoassays using the Human sCD14 and the Human LBP ELISA kits (Hycult Biotech, Uden, Netherlands). Serum cytokines and chemokines were measured by Luminex technology using the premixed Bio-Plex pro Human Cytokine Grp 1 panel 27-plex (IL-1β, IL-1ra, IL-2, IL-4, IL-5, IL-6, IL-7, IL-8, IL-9, IL-10, IL-12(p70), IL-13, IL-15, IL-17, basic FGF, eotaxin, G-CSF, GM-CSF, IFN-γ, IP-10, MCP-1, MIP-1α, MIP-1β, PGDF-BB, RANTES, TNF-α and VEGF) and the Bio-Plex Pro TGF-β Panel 3-plex (TGF-β 1, 2 and 3) assays (Bio-Rad Laboratories, CA). Acquisition was performed on the Bio Plex 200 platform (Bio Rad Laboratories).

### Statistical analysis

Statistical analysis was carried out in Statistica version 12 (StatSoft, OK) and GraphPad Prism version 5 software (GraphPad Software, CA). Normality of data was tested using the D’Agostino-Pearson omnibus test. Non-parametric data was described using medians and interquartile ranges. Differences between groups were compared using the Mann-Whitney test where there were only two groups or the Kruskal-Wallis test in cases of three or more groups. Multiple comparisons were preformed using the Dunn’s post-test where the Kruskal-Wallis *p* value was significant. Data with normal distribution was described using means and 95% confidence intervals (95% CI). Comparisons between groups (appearing as rows) were performed using the unpaired t-test when data was normally distributed. Categorical data was described using proportions and 95% CI and the differences in observed frequencies across groups were analysed using the χ2 test (or Fisher’s test when the number in a cell was low). Correlational analysis was performed using the Spearman rank correlation test. All hypothesis testing was done at 95% confidence intervals and results were regarded as significant if *p* < 0.05.

## Results

### Demographics

We recruited 169 patients into the study. The distribution of the patients was 46 HBV/HIV co-infected; 47 HBV mono-infected; 39 HIV mono-infected and; 37 HBV/HIV uninfected controls attending the gastroenterology clinic (Table 1).

**Table 1:** Demographic, immunologic and virologic characteristics of study participants according to group

	HBV/HIV (*n* = 46)	HBV (*n* = 47)	HIV (*n* = 39)	Controls (*n* = 37)	*p*
Age in years (mean ± standard deviation)	38.5 ± 8.2	37.9 ± 12.3	37.9 ± 9.7	43.8 ± 11.4	0.04
Males (%)	22 (47%)	22 (47%)	16 (39%)	25 (41%)	0.1
Ethnicity, *n* (%)					
African	31 (67%)	18 (38%)	17 (44%)	5(14%)	
Mixed	14 (32%)	25 (53%)	22 (56%)	30 (81%)	0.0001^b^
Caucasian	1 (1%)	3 (7%)	–	2 (5%)	
Asian	–	1 (2%)	–	–	
Body mass index	24.3 ± 5.1	28.0 ± 9.0	24.6 ± 3.5	27.0 ± 5.9	0.2
Alcohol consumption	8/46 (17%)	9/47 (19%)	11/39 (28%)	14/37 (38%)	0.1
Herbal medicine use (current/past)	1/46 (2%)	5/47 (11%)	1/39 (3%)	5/37 (14%)	0.11
ART, months Median (IQR)	36 (23–63)	n/a	36 (12–63)	n/a	
CD4 count, (cells/μL) Median (IQR)	328 (242–562)	922 (647–1297)	528 (367–657)	1031 (790–1215)	< 0.0001
CD4/CD8 ratio Median (IQR)	0.5 (0.3–0.7)	1.5 (1.1–2.1)	0.7 (0.6–1.0)	1.6 (1.3–2.1)	< 0.0001
CD4 nadir	256 (144–369)		281 (169–440)		0.2
Detectable plasma HIV-1 viral load	10/36 (27.8%)	n/a	6/33 (18.1%)	n/a	0.4
HIV-1 viral load > 1000 copies/ml^a^	6/36 (17%)	n/a	3/33 (9.1%)	n/a	0.5
Detectable plasma HBV DNA	22/45 (49%)	32/44 (73%)	n/a	n/a	0.03
Plasma HBV DNA > 2000 IU/ml^c^	12/45 (26%)	15/44 (32%)	n/a	n/a	0.5
HBeAg positive, (%)	13/46 (28%)	6/46 (13%)	n/a	n/a	0.1

^a^1000 copies/ml chosen for HIV-1 viral load as this defines virological failure in patients on ART

^b^Indicates where a chi test was used for statistical analysis

^c^HBV DNA > 2000 IU/ml cut-off used as it may indicate the need for therapy in some patients

### Immunological and virological characteristics

All co-infected and HIV mono-infected patients were on antiretroviraltherapy for ≥3 months (Table 1). All HIV-infected patients (HBV/HIV co-infected and HIV mono-infected) were on tenofovir together with emtricitabine or lamivudine. HIV virological suppression was achieved by 30/36 (78%) of co-infected compared to 27/33 (82%) of HIV mono-infected patients (Table 1). Only 12/47 (25%) of HBV mono-infected patients were on antiviral therapy while the duration of therapy varied from one week to 24 months. Of the 12 HBV patients receiving antiviral therapy, 11 were on tenofovir while a single patient was on lamivudine. The median CD4 cell count in the co-infected group was 328 cells/μl, IQR 242–562 compared to 528 cells/μl; IQR 367–657 in the HIV mono-infected group, *p* = 0.04. HBV viral loads had significant negative correlation with duration of therapy among co-infected patients, rho = − 0.48; *p* < 0.05.

### Prevalence of liver fibrosis using non-invasive markers

Due to the low number of HBV-mono-infected patients on therapy, statistical analysis was done taking the treated and untreated HBV mono-infected patients as a single group. The median of TE in the treated HBV mono-infected group was 7.2 kPa (IQR: 5.3–13.0) compared to 7.3 (IQR: 5.7–8.8) in the untreated HBV mono-infected, *p* = 0.98. Taken as a single group, the HBV mono-infected group had the highest proportion 7/38 (18%) of patients with TE values ≥13 kPa compared to 4/36 (11%) in the co-infected group and 3/32 (9%) in the HIV mono-infected group. (Table 2). The proportion of patients with TE values between 7 and 13 kPa was comparable between the co-infected and HBV-mono-infected groups at 11/36 (31%) and 12/38 (32%), respectively. These proportions were higher than the 1/32 (3%) and 6/29 (21%) among HIV-monoinfected and controls, respectively.

**Table 2:** Summary and distribution of fibrosis results according to group

	HBV/HIV	HBV	HIV	Controls	p
Valid Fibroscan	36/40 (90%)	38/41 (93%)	32/37 (86%)	29/30 (97%)	0.5
Fibroscan (kPa)	*n* = 36	**n** **= 38**	*n* = 32	*n* = 29	
< 7.0	21 (58%)	19 (50%)	28 (88%)	23 (79%)	
7.0–13	11 (31%)	12 (32%)	1 (3%)	6 (21%)	0.002^b^
≥13	4 (11%)	7 (18%)	3 (9%)	–	
APRI	*n* = 43	*n* = 35	*n* = 26	*n* = 22	
< 0.5	31 (72%)	25 (71%)	21 (81%)	19 (86%)	
0.5–1.5	9 (21%)	7 (20%)	4 (15%)	3 (14%)	0.8^b^
> 1.5	3 (7%)	3 (9%)	1 (4%)	–	
FIB-4	*n* = 42	*n* = 34	*n* = 26	*n* = 22	
< 1.30	31 (74%)	27 (79%)	19 (73%)	20 (91%)	
1.30–3.25	9 (21%)	4 (12%)	7 (27%)	2 (9%)	0.32^b^
> 3.25	2 (5%)	3 (9%)	–	–	
ALT, U/L^a^	34 (24–45)	25 (22–47)	31 (21–44)	24 (19–39)	0.3
AST, U/L^a^	31 (25–46)	27 (23–42)	32 (27–45)	21 (18–32)	0.001
Fibroscan, kPa^a^	6.2 (4.8–8.0)	6.9 (5.5–8.8)	4.7 (3.9–5.9)	5.6 (4.6–6.8)	0.001
APRI^a^	0.32 (0.24–0.58)	0.34 (0.21–0.66)	0.32 (0.23–0.46)	0.21 (0.13–0.42)	0.08
FIB-4^a^	0.96 (0.58–1.30)	0.86 (0.59–1.20)	0.77 (0.55–1.30)	0.63 (0.41–1.00)	0.1

^a^Median values and IQRs are shown in parenthesis

^b^Indicates where a chi test was used

The data in bold are entries where there were zero observations for the particular cell

### Microbial translocation markers

**Fig. 1:**
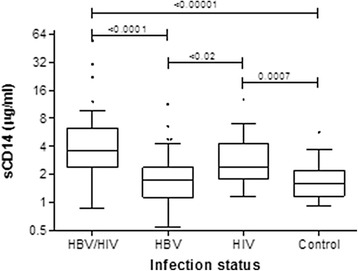
Plasma sCD14 according to group. The middle lines show the median while the boxes represent the 25th and 75th percentiles. The whiskers show the non-outlier range, asterix represent the outliers and the solid black squares indicate the extreme values. The y axis showing the sCD14 values is drawn using a logarithmic scale. The medians and interquartile ranges in parenthesis were as follows: Co-infected 3.6 μg/ml (IQR, 2.4–6.2); HBV mono-infected 1.8 μg/ml (1.1–2.4); HIV mono-infected 2.4 μg/ml (1.8–4.3) and control 1.6 (1.2–2.2)

The HBV/HIV group had median serum sCD14 concentration of 3.6 μg/ml; IQR 2.4–6.2 compared to 1.8 μg/ml; IQR 1.1–2.4 in the HBV group, 2.4 μg/ml; IQR 1.8–4.3 in the HIV group and 1.6 μg/ml; IQR 1.2–2.2 in the control group, *p* < 0.0001. Statistically significant differences in serum concentration were observed between co-infected compared to the HBV mono-infected (*p* < 0.0001); co-infected against the controls (*p* < 0.00001), HBV mono-infected versus HIV mono-infected (*p* = 0.02) and controls compared to HIV mono-infected (*p* = 0.0007) (Fig. 1). Serum LBP concentrations showed a trend toward increased levels in the co-infected group compared to the other groups, *p* = 0.06 (Additional file 1: Table S1). There were no statistically significant differences in the serum concentration of microbial translocation markers between the treated and untreated HBV mono-infected patients (data not shown).

### Expression of CD38, HLA-DR, PD-1 and CTLA-4 on CD4+ and CD8+ T lymphocytes

There were no significant differences in the expression of cell-associated immune markers between the treated and untreated HBV mono-infected groups and as a result were considered as a single group for statistical analysis. The median percentage CD8+/CD38+/HLA-DR+ co-expression in HBV/HIV co-infected patients was 30%; IQR 17–53 compared to 23%; IQR 16–33 in the HIV mono-infected group, 17%; IQR 14–22 in the HBV mono-infected and 16%; IQR 8–26 in the control group (*p* < 0.0001) (Table 3). The expression of PD-1 on both CD4+ and CD8+ T lymphocytes was also highest amongst co-infected patients compared to all other groups, *p* < 0.0001 in each instance. Post-test analysis showed that %CD8+/PD-1 was significantly higher among co-infected patients compared to HBV mono-infected and controls. Percentage CD4+/PD-1+ was significantly higher in the co-infected group (median 22; IQR 15–33) and HIV mono-infected (22; IQR 15–26) compared to HBV mono-infected (13.2; IQR 11–20) and controls (15; IQR 9–21). The HIV mono-infected group had higher %CD4+/PD-1+ compared to the control group (Table 3).

**Table 3:** Expression of cell-surface expressed markers on CD4+ and CD8+ T lymphocytes

	Co-infected	HBV Mono	HIV Mono	Control	p
%CD4/CD38+/HLA-DR+	9.9 (6.2–17)	4.7 (3.6–6.3)	7 (4.5–12)	3.5 (2.6–6)	< 0.0001
%CD8/CD38+/HLA-DR+	30 (17–53)	17 (14–22)	23 (16–33)	16 (7.7–26)	< 0.0001
%CD4+/PD-1+	25 (18–34)	18 (13–23)	21 (15–27)	13 (8–22)	< 0.0001
%CD8+/PD-1+	22 (15–33)	13.2 (11–20)	22 (15–26)	15 (9–21)	< 0.0001

Median and IQR (in parenthesis) are shown. Values are percentages of either CD4+ or CD8+ T cells expressing marker of interest

### Detection of serum cytokines

**Fig. 2:**
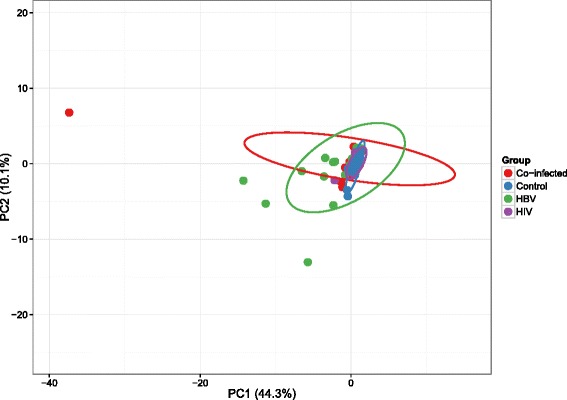
Principle component analysis of plasma concentrations of microbial translocation markers and cytokines. The plot shoes serum cytokine signatures of the four patients groups. The prediction ellipses represent 0.95 probability that a new observation from the same group will fall inside area. The ellipses for the co-infected and HBV groups appear to be distinct from those of the HIV mono-infected and control groups. The x and y-axis show principal component 1 and principal component 2 that explain 44.3% and 10.1% of the total variance observed, respectively

Within the HBV mono-infected group, only PDGF was statistically significantly different in concentration between the treated and untreated patients with the treated having median 3.84 ng/ml (IQR: 3.28–4.78) compared to 2.97 ng/ml (IQR: 1.81–3.57) among untreated patients, *p* = 0.04. We therefore considered the treated and untreated HBV mono-infected patients as a single group for multigroup comparison. Levels of basic fibroblast growth factor, IL-9 and IL-17 were particularly elevated in the HBV mono-infected group (Table 4). Serum levels of IP-10 were high among co-infected patients. TGF-beta isotypes were highly expressed in the HIV-mono-infected group. Analysis of serum cytokine concentrations was also performed excluding the control group significance levels (shown as p2 in Table 4). Correlation was analysed for all study participants as a single group as well as according to their infection status using all continuous variables measured in the study. There were significant positive correlations between IP-10 and sCD14 (rho = 0.32; *p* = 0.00008) and IP-10 with LBP (rho = 0.19; *p* = 0.02). IP-10 was also positively correlated with HIV viral load (rho = 0.42; *p* = 0.004) and HBV viral load (rho = 0.37, *p* = 0.02) in the co-infected group. Principle component analysis of serum cytokine concentrations showed relatively good distinction between HBV mono-infected, HBV/HIV co-infected patients from the HIV mono-infected and controls. There was poor distinction between the HIV mono-infected participants and controls (Fig. 2).

**Table 4:** Median plasma cytokine concentrations

	Units	LDL	HBV/HIV, *n* = 45	HBV, *n* = 44	HIV, *n* = 39	Control, *n* = 19	p	p2
IL-1β	pg/ml	0.47	1.3 (0.9–2.8)	2 (1.5–3.1)	1.2 (0.9–1.9)	1.7 (1.2–2.8)	*0.001*	*0.0004*
IL-1ra	pg/ml	5.33	59 (40.4–100.8)	110.8 (85.1–376.7)	62.2 (40.4–85.8)	69.8 (53.7–106.3)	*< 0.00001*	*< 0.00001*
IL-2	pg/ml	1.52	0 (0–5.4)	8 (0.5–18.4)	0 (0–7.3)	0 (0–4.7)	*0.0002*	*0.0002*
IL-4	pg/ml	0.24	2.2 (1.6–3)	2.8 (2.1–4.1)	2.2 (1.6–3.2)	2.3 (2–2.9)	*0.03*	*0.02*
IL-5^a^	pg/ml	1.00	0 (0–7)	0 (0–15.5)	0 (0–0)	0 (0–4)	*n/a*	*n/a*
IL-6	pg/ml	1.68	10 (0.8–18.3)	11.3 (7.2–29.3)	4.3 (0.5–10.3)	6.3 (1.5–12.5)	*0.002*	*0.005*
IL-7	pg/ml	0.84	9 (3.7–14.6)	7.4 (2.5–14.8)	1.2 (0–7.9)	5.9 (1.2–9)	*0.002*	*0.001*
IL-8	pg/ml	2.44	26.8 (19.5–53.3)	51.9 (22.8–121.8)	21.7 (13.9–43.6)	120.1 (35.2–437.2)	*< 0.00001*	*0.002*
IL-9	pg/ml	1.19	54.7 (44.5–73.8)	73.1 (55.6–118.5)	59.5 (45.5–83.7)	78.2 (57.6–97.1)	*0.01*	*0.02*
IL-10	pg/ml	2.21	10.2 (5.8–18.8)	15.9 (11.3–29.2)	9.5 (5.6–14.5)	9.7 (7.7–15.9)	*0.001*	*0.005*
IL-12	pg/ml	2.25	50.8 (31.9–63.6)	47.4 (27.1–87.9)	35.2 (19.4–59.9)	44.5 (31.7–65.4)	0.3	*0.2*
IL-13	pg/ml	0.40	12 (6.2–24.8)	13.8 (8–29.6)	9.5 (5.2–13.7)	6.5 (4.8–10.4)	*0.004*	*0.02*
IL-15^a^	pg/ml	5.73	0 (0–0)	0 (0–0)	0 (0–0)	0 (0–0)	*n/a*	
IL-17	pg/ml	5.81	44.5 (27.7–84.3)	81.7 (61.6–133.2)	67.7 (54.1–111.9)	78.5 (64.1–110.4)	*0.004*	*0.006*
EOTAXIN	pg/ml	1.75	107.9 (81.2–172.8)	129.3 (72.1–171.3)	115.8 (73.9–160.1)	142.5 (93.1–261.1)	0.4	*0.8*
Basic-FGF	pg/ml	3.91	43.1 (21.8–66.9)	65.7 (46.2–92.3)	44.8 (24.2–61.3)	50.4 (34.2–65.7)	*0.0006*	*0.0002*
G-CSF	pg/ml	3.06	51.1 (32.1–66.1)	59.7 (44.1–98.6)	41.9 (30.3–58.1)	37.6 (32.1–66.1)	*0.006*	*0.004*
GM-CSF	pg/ml	2.9	0 (0–22.2)	25.5 (0–87.6)	0 (0–35.7)	0.6 (0–15.3)	*0.006*	*0.003*
IFN-γ	pg/ml	42.9	88.7 (57.5–145.3)	121.2 (80.1–182.0)	66.7 (48–125.7)	97.2 (51.1–116.7)	*0.004*	*0.004*
IP-10	ng/ml	4.94	1.5 (1.0–2.8)	0.9 (0.7–1.5)	1.1 (0.8–1.5)	1 (0.8–1.2)	*0.0001*	*0.0001*
MCP-1	pg/ml	1.24	4.7 (0–27.2)	21.3 (0–54.4)	0.4 (0–20.5)	17.3 (0–78.8)	*0.03*	*0.2*
MIP-1a	pg/ml	0.15	4.4 (1.3–7.7)	7.3 (4.6–11.4)	4.7 (2.5–6.3)	8.2 (5.3–10.3)	*0.001*	*0.006*
PDGF	ng/ml	1.00	2.4 (1.7–3.3)	3.2 (1.8–3.9)	2.9 (1.9–3.8)	3.3 (2.3–4.5)	0.09	*0.2*
MIP-1b	pg/ml	0.46	140.9 (95.5–218.8)	147.3 (102.7–199.1)	128.8 (103.8–227.4)	217 (155.5–264.7)	0.06	*0.9*
RANTES	ng/ml	3.72	19.9 (16.7–26.7)	19.0 (13.8–23.1)	19.1 (16.7–22.6)	22.1 (17.8–24.7)	0.2	*0.3*
TNF-a	pg/ml	3.77	19.5 (15.1–27.4)	23.5 (18.5–54.5)	19.5 (15–24)	18.5 (15.1–26.2)	*0.01*	*0.007*
VEGF	pg/ml	2.30	88.9 (52.2–147.7)	117.7 (60–211.5)	77.8 (45.2–138)	89.6 (51.5–160.5)	0.2	*0.09*
TGF-β1	ng/ml		41.7 (34.1–53.6)	46.4 (38.3–56.8)	49.7 (42.1–55.7)	48.2 (42.5–55)	0.10	*0.06*
TGF-β2	ng/ml		1.8 (1.7–1.9)	2.1 (1.8–2.4)	2.3 (1.8–2.5)	2 (1.7–2.4)	*< 0.00001*	*< 0.00001*
TGF-β3	ng/ml		1.3(1.2–1.5)	1.9 (1.4–2.2)	2.2 (1.5–2.3)	1.8 (1.4–2.2)	*< 0.00001*	*< 0.00001*

^a^Plasma levels of IL-5 and IL-15 were below detection limit in most samples across the groups hence the medians of 0 pg/ml. Column heading p2 shows the level of significance of Kruskal-Wallis test excluding the control group

### Correlation of viral and immune markers with non-invasive tests of liver fibrosis

Correlation was performed for all participants without stratification of groups and according to the individual groups. In the ungrouped analyses, median stiffness had significant positive correlation with HIV viral load (rho = 0.29, *p* = 0.02) and HBV viral load (rho = 0.30, *p* = 0.007). In addition, HBV viral load was significantly correlated with APRI score within the HBV mono-infected group (rho = 0.5, *p* = 0.003). Soluble CD14 positively correlated with %CD8+/CD38+/HLA-DR+ (rho = 0.19, *p* = 0.01) and also percentage CD8+/PD-1+ expression (rho = 0.2, *p* = 0.009). Soluble CD14 showed significant negative correlation to CD4+ T cell count (rho = − 0.04, *p* < 0.0001) and CD4/CD8 ratio (rho = − 0.5, *p* < 0.00001) (Additional file 1: Table S3). HIV viral loads positively correlated with CD8+/CD38+/HLA-DR+ expression within the co-infected group (rho = 0.4, *p* < 0.05) but not in the HIV mono-infected (Additional file 1: Figure S2). We did not observe any statistically significant correlations between cytokine levels and liver stiffness except for IL-5 (rho = 0.17, *p* = 0.049), TGF-β2 (rho = − 0.26, *p* = 0.003), TGF-β2 (rho = − 0.31, *p* < 0.001) and TGF-β3 (rho = − 0.31, *p* < 0.001) (Additional file 1: Table S3).

### Molecular characterization of HBV and association with liver fibrosis

Of 22 viraemic co-infected patients, 13 (59.1%) were successfully sequenced compared to 29/32 (90.6%) of HBV mono-infected participants (Additional file 1: Figure S3). The medians of the TE scores for the different genotypes were 7.9 kPa; IQR 5.8–12.1 for genotype A (*n* = 21), 6.3 kPa; IQR 5.8–7.6 for genotype D (*n* = 11) and 7.0 kPa; IQR 5.3–7.9 for genotype E (*n* = 4). There was no statistically significant difference in TE scores according to the HBV genotype, *p* = 0.43 (Additional file 1: Table S2). No classic mutations associated with resistance to nucleotide reverse transcriptase inhibitors were observed in the co-infected or HBV mono-infected group.

## Discussion

HIV co-infection has an adverse impact on the natural progression of hepatitis B [16]. On the contrary, data describing the effect of hepatitis B on HIV infection is limited. Despite being on ART, co-infected patients had lower CD4 cell counts compared to those infected with HIV alone. The proportion of patients with plasma HIV viral load > 1000 copies/ml was also higher among those with co-infection (17%) compared to those with HIV alone (9%) as shown in Table 2. This may also explain the low CD4 cell counts among co-infected patients as negative correlation was observed between HIV viral load and CD4 cell counts. We did not have the data on the CD4 counts before these patients were started on ART but other studies have shown that HBV/HIV co-infected individuals have lower CD4 counts or impaired CD4 cell count recovery compared to those with resolved HBV infection and HBV-uninfected patients independent of duration of ART [4, 17–19]. Data on nadir CD4 counts in this study showed no statistically significant differences between the co-infected and HIV mono-infected groups. Reasons for the decreased immune recovery include upregulation of apoptotic pathways in co-infection and sequestration of lymphocytes within the spleen following HBV-driven liver fibrosis [19, 20]. Low CD4/CD8 ratios despite effective ART are associated with continuous immune dysregulation, immune senescence and are predictive of non-AIDS morbidity and mortality [21]. Our results show co-infected patients with significantly lower median CD4/CD8 ratio compared to those infected with HIV alone which is explained by the fact that normalisation of CD4/CD8 ratio is solely based on the recovery of CD4 cells.

HBV mono-infected patients more frequently had detectable HBV DNA compared to the co-infected patients; (49% vs 73%, *p* = 0.03). This is expected as not all the HBV mono-infected patients were on therapy. The presence of HBV DNA among the co-infected patients who were on effective therapy may indicate non-adherence to therapy. Another reason may be that HBV DNA load on therapy is a function of time because of the significant negative correlation observed between time on therapy and HBV viraemia. A study of Zambian and South African patients reported that only 15/21 (71%) of co-infected patients achieved HBV suppression after a year of tenofovir plus lamivudine or emtricitabine [22]. However, not all HBV-infected patients on therapy will achieve virologic suppression even without obvious drug-resistance mutations, especially those with high levels of replication such as in HIV co-infection and those that are HBeAg positive have delayed viral suppression [23–25]. The higher prevalence of HBeAg among co-infected patients compared to HBV mono-infected patients is a well-recognised effect of HIV infection [16, 26–28]. Even with administration of an effective regimen such as tenofovir plus emtricitabine, not all patients will achieve HBeAg seroconversion. A previous study reported that after 5 years of continuous therapy HBeAg seroconversion was achieved by only 57% of HIV/HBV co-infected patients [29].

Our results of TE showed that the HBV mono-infected group had the highest proportion of patients with significant or advanced fibrosis and the highest the median of TE value compared to all other groups. The results showing a higher proportion of patients with significant fibrosis among those with HBV/HIV coinfection compared to HIV mono-infected patients are consistent with findings from other groups where coinfection was associated with higher TE values [30–32]. HIV alone independently increases liver fibrosis progression rates [33, 34]. However, the finding of a higher proportion of HBV mono-infected individuals with advanced fibrosis compared to HIV mono-infected is not unexpected because the majority of HBV mono-infected patients in this cohort were not on treatment. Treatment of hepatitis B improves liver fibrosis and this probably explains the reason for lower TE values in the co-infected patients compared to those with HBV mono-infection [35].

Soluble CD14 is a marker of monocyte/macrophage activation that is normally induced by exposure to LPS. The results from this study showed that co-infected patients had the highest plasma sCD14 levels compared to those infected with either HBV or HIV alone. Research in paediatric and adult patients infected with HIV has shown that despite ART, there is persistently higher immune activation punctuated by elevated levels of sCD14 and that this is not related to HIV viraemia [36, 37]. Our results also support these findings because plasma sCD14 did not correlate with either plasma HBV DNA or HIV RNA, thus supporting the idea that gut microbial translocation is independent of on-treatment viral load. This may explain why no difference was detected in microbial translocation markers between HBV mono-infected patients that were on therapy and those that were not. A potential mechanism for continued monocyte activation may be due to blunted recovery of CD4 T cells during ART [38]. However, this study cannot determine the rate of CD4 T cell recovery of the HIV-infected patients that were recruited because of its cross-sectional design. ART does not restore gut integrity and therefore continued elevations of LBP and sCD14 are expected [39, 40]. The overall finding from the microbial translocation markers and liver fibrosis markers would seem to suggest that gut translocation is not associated with liver stiffness in this treated cohort. Instead, according to the results of the ungrouped analysis viral replication is the driver of liver fibrosis.

Co-infected patients displayed the highest percentage of CD4+ and CD8+ T lymphocytes co-expressing HLA-DR and CD38 compared to other groups in this study. The results suggest that HIV infection is the driver of immune activation because of the positive correlation between HIV viraemia and CD8/CD38/HLA-DR expression. Significant positive correlation was observed in the ungrouped analysis and the co-infected group. Interestingly, there was no correlation between the expression of peripheral blood cell-based immune markers of activation and liver stiffness as measured by any of the fibrosis markers. This may be due to the fact that the study measured immune activation in peripheral blood and not the hepatic environment. Research on the hepatic environment in HBV/HIV co-infected patients previously found no evidence of increased inflammation but rather apoptosis as the driver of liver fibrosis [41]. The elevated serum concentrations of IL-9, IL-17 and fibrogenic basic fibroblast growth factor in plasma of HBV mono-infected patients support recent reports highlighting the roles of these biomarkers in pathogenesis of liver fibrosis due to viral hepatitis [42–45]. The co-infected group had significantly higher levels of IP-10 compared to the HBV mono-infected group. Increased IP-10 levels could be an indicator of continued immune activation from viral replication and gut translocation in HBV/HIV co-infection and have been described as remaining elevated in co-infected patients despite combination antiretroviral therapy [46].

Our study hypothesis was that in co-infection with HIV and HBV, HIV facilitates the development of liver fibrosis via immune activation in response to the leakage of gut microbial products into the systemic circulation. Instead, we found that although there was increased microbial translocation shown by increased sCD14 and elevated immune dysregulation in HBV/HIV co-infected patients, the proportion of patients with moderate/advanced liver fibrosis as determined by TE was higher in the HBV mono-infected group compared to those with co-infection. The finding of no significant correlation between microbial translocation markers and liver fibrosis scores would seem to imply that microbial products are not the driver of fibrosis but rather viral replication. This is because TE had significant positive correlation with both HIV and HBV viraemia in the ungrouped analysis. The relationship of active viral replication with fibrosis implicates active antigen-specific anti-viral responses as being important to pathogenesis of liver fibrosis. Viral replication as the driver of liver fibrosis has been reported among treatment-naïve HBV/HIV co-infected patients in Zambia and Switzerland [47].

There are some limitations in this study. The sample size of treated and untreated HBV mono-infected patients was too small to consider them as separate groups, which would have been ideal. The small number of HBV mono-infected patients on therapy is a reflection of the lack of prioritization of therapy for HBV infections in South Africa. The non-availability of liver biopsies from study participants makes it difficult to validate the accuracy of the results of the non-invasive tests for fibrosis although the use of Fibroscan has been validated in numerous studies in the past [48, 49]. Valid TE measurements were not obtained on all patients due to visceral obesity and unavailability of a large probe. An effect of other undiagnosed infections on expression of markers of immune activation cannot be eliminated although any patients with overt symptoms of other infectious diseases were excluded. Another limitation is that the assessment of immune markers was not virus-specific. This study did not measure microbial components such as LPS or microbial 16S rRNA per se but rather products of the host physiologic response to microbial translocation. We measured LBP and sCD14 in this current study because LPS has a short half-life in circulation and its measurement using the widely utilized Limulus assay is subject to interference from other molecules in the blood where it may be bound to serum binding proteins [50]. Other studies have shown strong correlation between LPS levels and sCD14 and/or LBP [50, 51]. The effect of previously described single nucleotide polymorphisms of the CD14 gene on the expression of sCD14 was not explored in this study but variations in the gene could potentially influence the long-term recovery of CD4 T cells in HIV-infected patients on HAART [52]. Nevertheless, the data from this study is important to the process of developing prospective studies to monitor prognosis of HBV infection, especially among HBV-mono-infected patients who are under-diagnosed and under-treated.

## Conclusions

The findings suggest that HBV-mono-infected patients in this study have worse liver fibrosis than patients with HBV/HIV co-infection despite immune activation being higher in the latter group. HBV/HIV co-infected patients in South Africa have better access to screening, effective treatment and monitoring than those that are HBV-mono-infected. There is a need for screening for HBV infection and monitoring of HBV mono-infected patients to allow initiation of timely and effective interventions in order to prevent the development of complications such as advanced liver fibrosis and/or hepatocellular carcinoma.

## Additional file

Additional file 1: **Table S1.** Serum LPB concentration according to patient group (μg/ml). **Table S2.** Proportions of fibrosis scores according to the HBV genotype. **Table S3.** Multiple correlation analysis of all patients ungrouped. Statistically significant correlations appear in red font. **Figure S1.** Gating strategy for Immune Activation panel. Plot A shows the singlet population gating while Plot B represents the SSC against FSC plot indicating the position of the singlet cells of the lymphocyte population. Plot C shows the lymphocyte population as shown by less complexity (SS) and intense staining for CD45-KO in a plot of side scatter against CD45-KO. Plot D shows the CD3+ population (Gate G) as gated from Gate F shown in Plot C. Using colour precedence and back gating, Plot D also shows the non-lymphocyte population (red colour) that is included within gate F based on use of complexity (SS INT) and staining for CD45. Picture E shows the CD4+ in the blue colour (gate H) and the CD8+ lymphocytes in the magenta colour (gate I). Plot F shows the CD8+ population staining for CD38-PE and HLA-DR-APC gated from gate I. The gate placement was based on defined fluorescence minus one (FMO) settings. **Figure S2.** Scatter plot of % CD8/CD38+/HLA-DR+ in the co-infected group. The plot only includes HBV/HIV co-infected patients. All patients with undetectable HIV viral load are assigned values of zero and appear on the y-axis as dots corresponding with the percentage expression CD8/CD38+/HLA-DR. Frequency of HBV genotypes according to HBV and HIV infection status. Among the 13 co-infected patients whose HBV was successfully sequenced, 8 (62%) were infected with HBV genotype A, 3 (23%) with D and 2 had HBV genotype E (15%). The distribution of genotypes among the HBV mono-infected patients was- 16/29 (55%) A, 11/29 (38%) D and 2/29 (7%) E. The red columns represent HBV genotype A, green is for genotype D and the blue corresponds to genotype E. Genotyping was frequently more successful in the HBV mono-infected group compared to the co-infected group. (DOCX 475 kb)
