# Supplementary material for: HBV and HIV viral load but not microbial translocation or immune activation are associated with liver fibrosis among patients in South Africa
Source: BMC Infect Dis. 2018 May 8;18:214. doi: 10.1186/s12879-018-3115-8 (PMC5941637; doi:10.1186/s12879-018-3115-8)
Supplement: Supplementary file 1 — Table S1. Serum LPB concentration according to patient group (μg/ml). Table S2. Proportions of fibrosis scores according to the HBV genotype. Table S3. Multiple correlation analysis of all patients ungrouped. Statistically significant correlations appear in red font. Figure S1. Gating strategy for Immune Activation panel. Plot A shows the singlet population gating while Plot B represents the SSC against FSC plot indicating the position of the singlet cells of the lymphocyte population. Plot C shows the lymphocyte population as shown by less complexity (SS) and intense staining for CD45-KO in a plot of side scatter against CD45-KO. Plot D shows the CD3+ population (Gate G) as gated from Gate F shown in Plot C. Using colour precedence and back gating, Plot D also shows the non-lymphocyte population (red colour) that is included within gate F based on use of complexity (SS INT) and staining for CD45. Picture E shows the CD4+ in the blue colour (gate H) and the CD8+ lymphocytes in the magenta colour (gate I). Plot F shows the CD8+ population staining for CD38-PE and HLA-DR-APC gated from gate I. The gate placement was based on defined fluorescence minus one (FMO) settings. Figure S2. Scatter plot of % CD8/CD38+/HLA-DR+ in the co-infected group. The plot only includes HBV/HIV co-infected patients. All patients with undetectable HIV viral load are assigned values of zero and appear on the y-axis as dots corresponding with the percentage expression CD8/CD38+/HLA-DR. Frequency of HBV genotypes according to HBV and HIV infection status. Among the 13 co-infected patients whose HBV was successfully sequenced, 8 (62%) were infected with HBV genotype A, 3 (23%) with D and 2 had HBV genotype E (15%). The distribution of genotypes among the HBV mono-infected patients was- 16/29 (55%) A, 11/29 (38%) D and 2/29 (7%) E. The red columns represent HBV genotype A, green is for genotype D and the blue corresponds to genotype E. Genotyping was frequently more successful in the HBV mono-in [file 12879_2018_3115_MOESM1_ESM.docx]

**Supplementary Material**

**Supplementary Table 1 Serum LPB concentration according to patient group (µg/ml)**

|  | Co-infected | HBV mono | HIV mono | Control | p |
| --- | --- | --- | --- | --- | --- |
| n | 42 | 38 | 38 | 37 |  |
| Median (IQR) | 12.4 (10.2-15.6) | 10.8 (8.4-13.4) | 9.9 (7.8-16.0) | 9.6 (8.2-13.3) | 0.1 |

**Supplementary Table 2: Proportions of fibrosis scores according to the HBV genotype.**

|  | <7.0 kPa | 7-13 kPa | >13 kPa |
| --- | --- | --- | --- |
| Genotype A, n=21 | 7 (33%) | 9 (43%) | 5 (24%) |
| Genotype D, n=11 | 7 (64%) | 3 (27%) | 1 (9%) |
| Genotype E, n=4 | 2 (50%) | 2 (50%) | 0 |

**Supplementary Table 3 Multiple correlation analysis of all patients ungrouped.** Statistically significant correlations appear in red font.

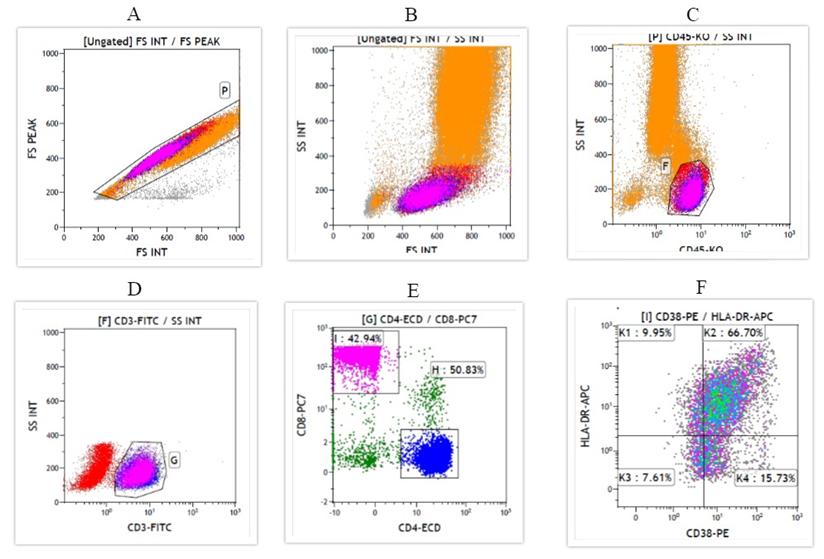


**Supplementary Figure 1:** Gating strategy for Immune Activation panel. Plot A shows the singlet population gating while Plot B represents the SSC against FSC plot indicating the position of the singlet cells of the lymphocyte population. Plot C shows the lymphocyte population as shown by less complexity (SS) and intense staining for CD45-KO in a plot of side scatter against CD45-KO. Plot D shows the CD3+ population (Gate G) as gated from Gate F shown in Plot C. Using colour precedence and back gating, Plot D also shows the non-lymphocyte population (red colour) that is included within gate F based on use of complexity (SS INT) and staining for CD45. Picture E shows the CD4+ in the blue colour (gate H) and the CD8+ lymphocytes in the magenta colour (gate I). Plot F shows the CD8+ population staining for CD38-PE and HLA-DR-APC gated from gate I. The gate placement was based on defined fluorescence minus one (FMO) settings.


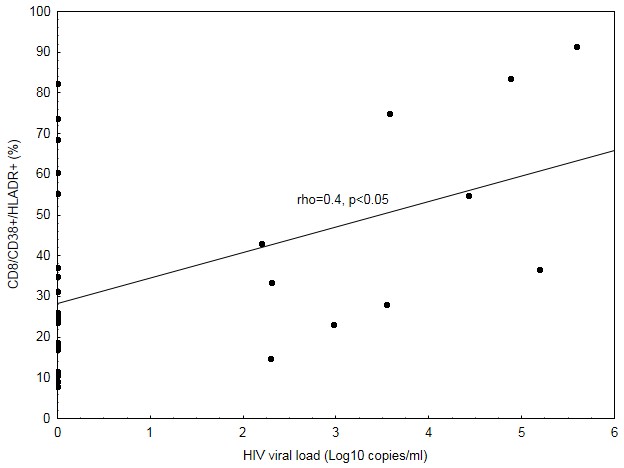


**Supplementary Figure 1 Scatter plot of % CD8/CD38+/HLA-DR+ in the co-infected group**. The plot only includes HBV/HIV co-infected patients. All patients with undetectable HIV viral load are assigned values of zero and appear on the y-axis as dots corresponding with the percentage expression CD8/CD38+/HLA-DR.

**Supplementary Figure 2 Frequency of HBV genotypes according to HBV and HIV infection status**. Among the 13 co-infected patients whose HBV was successfully sequenced, 8 (62%) were infected with HBV genotype A, 3 (23%) with D and 2 had HBV genotype E (15%). The distribution of genotypes among the HBV mono-infected patients was- 16/29 (55%) A, 11/29 (38%) D and 2/29 (7%) E**.** The red columns represent HBV genotype A, green is for genotype D and the blue corresponds to genotype E. Genotyping was frequently more successful in the HBV mono-infected group compared to the co-infected group.
